# Regulation of hnRNPA1 by microRNAs controls the miR-18a–*K-RAS* axis in chemotherapy-resistant ovarian cancer

**DOI:** 10.1038/celldisc.2017.29

**Published:** 2017-09-12

**Authors:** Cristian Rodriguez-Aguayo, Paloma del C Monroig, Roxana S Redis, Emine Bayraktar, Maria I Almeida, Cristina Ivan, Enrique Fuentes-Mattei, Mohammed H Rashed, Arturo Chavez-Reyes, Bulent Ozpolat, Rahul Mitra, Anil K Sood, George A Calin, Gabriel Lopez-Berestein

**Affiliations:** 1 Department of Experimental Therapeutics, The University of Texas MD Anderson Cancer Center, Houston, TX, USA; 2 Center for RNA Interference and Non-Coding RNA, The University of Texas MD Anderson Cancer Center, Houston, TX, USA; 3 Instituto de Investigação e Inovação em Saúde/Institute for Research and Innovation in Health (I3S) and Instituto de Engenharia Biomédica (INEB), University of Porto, Porto, Portugal; 4 Department of Pharmacology and Toxicology, Faculty of Pharmacy, The University of Al-Azhar, Cairo, Egypt; 5 Center for Research and Advanced Studies, National Polytechnic Institute (CINVESTAV del IPN), Monterrey, Mexico; 6 Department of Gynecologic Oncology, The University of Texas MD Anderson Cancer Center, Houston, TX, USA; 7 Department of Cancer Biology, The University of Texas MD Anderson Cancer Center, Houston, TX, USA

**Keywords:** chemotherapy resistance, hnRNPA1, K-RAS, microRNAs, ovarian cancer, RNA-binding proteins

## Abstract

The regulation of microRNA (miRNA) biogenesis, function and degradation involves a range of mechanisms, including interactions with RNA-binding proteins. The potential contribution of regulatory miRNAs to the expression of these RNA interactor proteins that could control other miRNAs expression is still unclear. Here we demonstrate a regulatory circuit involving oncogenic and tumor-suppressor miRNAs and an RNA-binding protein in a chemotherapy-resistant ovarian cancer model. We identified and characterized miR-15a-5p and miR-25-3p as negative regulators of hnRNPA1 expression, which is required for the processing of miR-18a-3p, an inhibitor of the *K-RAS* oncogene. The inhibition of miR-25-3p and miR-15a-5p decreased the proliferation, motility, invasiveness and angiogenic potential and increased apoptosis when combined with docetaxel. Alteration of this regulatory circuit causes poor overall survival outcome in ovarian cancer patients. These results highlight miR-15a-5p and miR-25-3p as key regulators of miR-18a-3p expression and its downstream target *K-RAS*, through direct modulation of hnRNPA1 expression. Our results demonstrate the therapeutic potential of inhibiting miR-25-3p and miR-15a-5p and the use of miR-18a-3p/KRAS ratio as a prominent outcome prognostic factor.

## Introduction

Regulation of the hnRNPA1 RNA-binding protein by either miR-15a-5p or miR-25-3p leads to increased tumor growth by inhibiting the biogenesis of miR-18a-3p, an inhibitor of the *K-RAS* oncogene. This work identifies miR-15a-5p/miR-25-3p/miR-18a-3p/*K-RAS* pathway as a potential target for overcoming resistance to chemotherapy in cancer cells and improving patient outcomes.

MicroRNAs (miRNAs) are small non-coding RNAs that repress gene expression [1]; they target most protein-coding transcripts and are involved in nearly all developmental and pathological processes in animals [2]. The biogenesis of miRNAs is under tight temporal and spatial control, and their dysregulation is associated with many human diseases, which makes them important targets for therapy, particularly in cancer [3]. The overall dysregulation of miRNA expression is an emerging feature in cancer and alterations of a specific miRNAs may be tumor-type specific [4–6]. However, miRNA deregulation is caused not only by gene amplification or disruption [7] but also is most likely related to changes in transcription rate, processing and activity. Although transcription factors and chromatin modulators account for most alterations in miRNA production [8], explaining many cases of miRNA overexpression in cancer [9, 10], it has become apparent that miRNA regulators themselves are subject to a sophisticated control. Several studies have reported that RNA-binding proteins are key components in the determination of miRNA function [11, 12], as they control different stages of miRNA biogenesis and their localization, degradation and activity [13]. Alteration of RNA-binding protein function can lead to impairment in any of the crucial steps of the miRNA pathway [14].

It has been suggested that the RNA-binding protein hnRNPA1 is required for processing of miR-18a and facilitates its Drosha-mediated processing [15]. Processing of the miR-18a precursor stem-loop produces two mature miRNAs: miR-18a (miR-18a-5p) and miR-18a* (miR-18a-3p) [15]. miR-18a-3p has been shown to function as a tumor suppressor by targeting *K-RAS* [16]. Also, it has been described that hnRNP A1 binds to let-7a and interferes with the binding of KSRP, which is known to promote let-7a biogenesis [17]. MiRNAs and RNA-binding proteins interplay under several physiological conditions or in response to external stimuli [18]. The mechanisms underlying the regulation of hnRNPA1 expression and their role in cancer progression are not well understood. We hypothesize that there is a circuit of miRNAs’ regulation between oncogenic and tumor-suppressor miRNAs, through direct modulation of hnRNPA1 expression. We investigated the interactions of miR-25-3p and miR-15a-5p and their relevance for miR-18a–*K-RAS* axis in chemotherapy-resistant ovarian cancer. The potential implications of the findings demonstrate the importance of miRNA circuits’ regulation and to evaluate their roles as therapeutics target.

## Results

### hnRNPA1 expression is decreased in chemoresistant ovarian cancer cells

**Figure 1:**
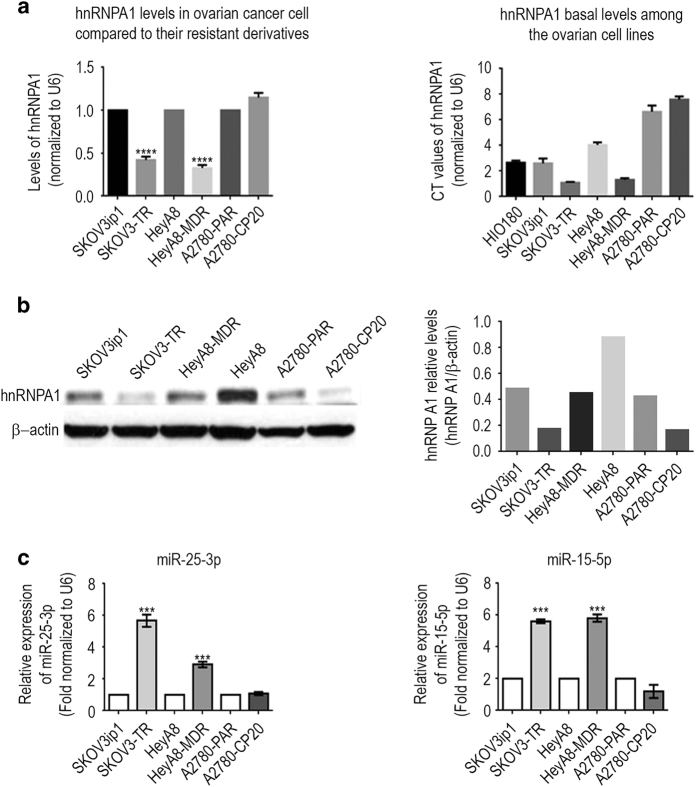
miR-25-3p and miR-15a-5p are overexpressed and inversely associated with hnRNPA1 expression level in chemotherapy-resistant ovarian cancer cell lines. (**a**) *hnRNPA1* mRNA expression was elevated in chemotherapy-sensitive parental ovarian cancer cell lines, compared with its resistant derivatives. Right panel shows the *hnRNPA1* basal levels among the ovarian cell lines. Total RNA isolated from human ovarian epithelial cancer cell lines was subjected to quantitative PCR (qPCR) analysis for *hnRNPA1* and *U6* by using validated Cyber assays. Data are presented as mean±s.e.m. *P<0.05; **P<0.01; ***P<0.001; ****P<0.0001. (**b**) hnRNPA1 protein levels were decreased in taxane-resistant ovarian cancer cell lines. Whole-cell lysates isolated from human ovarian cancer cell lines were subjected to western blot analysis for hnRNPA1 and β-actin. (**c**) miR-25-3p and miR-15a-5p expression levels were higher in taxane-resistant ovarian cancer cell lines than in sensitive or cisplatin-resistant cell lines. qPCR analysis of miR-25-3p and miR-15a-5p in ovarian cancer cell lines was performed using validated TaqMan assays. Data are presented as means±s.e.m. *P<0.05; **P<0.01; ***P<0.001; ****P<0.0001.

To analyze the physiological relevance of hnRNPA1 expression in chemoresistant ovarian cancer, we performed a screen for protein expression in three chemotherapy-sensitive (parental sensitives: SKOV3IP1, HeyA8, and A2780) and resistant pairs of ovarian cancer cell lines (resistants: SKOV3-TR, HeyA8-MDR, and A2780-CP20) (Figure 1a). Although the expression of hnRNPA1 was lower in SKOV3-TR (0.420-folds) and HeyA8-MDR (0.326-folds) resistant cells than in their parental cells, the expression of A2780-CP20 was the same as in its parental cells. This was confirmed by western blotting although the mRNA levels were not correlated with the protein levels in A2780-CP20 (Figure 1b).

We next performed computational analyses to predict which miRNAs might target the human *hnRNPA1* gene using four widely applied miRNA target prediction methods: Miranda [19], TargetScan [20], PicTar [21], and RNA22 [22]. By using these prediction methods with relaxed stringencies, we maximized the number of miRNAs chosen for validation to avoid missing those that target *hnRNPA1*. Altogether, 42 miRNAs were predicted by at least one of these methods to target the *hnRNPA1* 3′-untranslated region (3′-UTR). Only four (miR-149, miR-92, miR-25-3p and miR-15a-5p) of these 42 miRNAs were expressed at a higher level in the resistant cell line than in the parental cell line in at least one cell line pair, with a cutoff of 0.25-fold change (Figure 1c; Supplementary Figure S1), and therefore we decided to further investigate these miRNA. Real-time PCR was performed to determine which of the miRNAs predicted to target *hnRNPA1* were overexpressed in the resistant cell lines (Figure 1a). Three miRNAs (miR-25-3p, miR-15a-5p and miR-92) were expressed at a higher level in the resistant cells in at least two cell line pairs, and two of those (miR-25-3p and miR-15a-5p) were expressed at significantly higher levels (sixfold and threefold, respectively) in resistant cells lines than in parental cell lines, thus emerging as putative regulators of *hnRNPA1* (Figure 1c). We selected the chemoresistant ovarian cancer cell lines SKOV3-TR and HeyA8-MDR for further studies because of their high *hnRNPA1* expression (Figure 1a) and *in vivo* tumorigenicity.

### hnRNPA1 is a direct target of miR-15a-5p and miR-25-3p

hnRNPA1 has been shown to be a multifunctional RNA-binding protein required for the processing of miR-18a in the nucleus for Drosha-mediated processing. miR-18a-3p targets the *K-RAS* oncogene and thus serves as a tumor suppressor. We therefore carried out a comparative analysis of Drosha, hnRNPA1 and *K-RAS* expression, using the Affymetrix platform RNA Array and western blotting in HeyA8 and HeyA8-MDR ovarian cancer cell lines. Expression of hnRNPA1, Drosha and *K-RAS* in HeyA8 cells was inversely correlated with their expression in HeyA8-MDR (Supplementary Figure S2a–c). The observation that low hnRNPA1 expression was correlated with high *K-RAS* expression prompted us to elucidate how the biological functions of the hnRNPA1 protein are affected by miRNAs to better understand the protein’s role in ovarian cancer.

**Figure 2:**
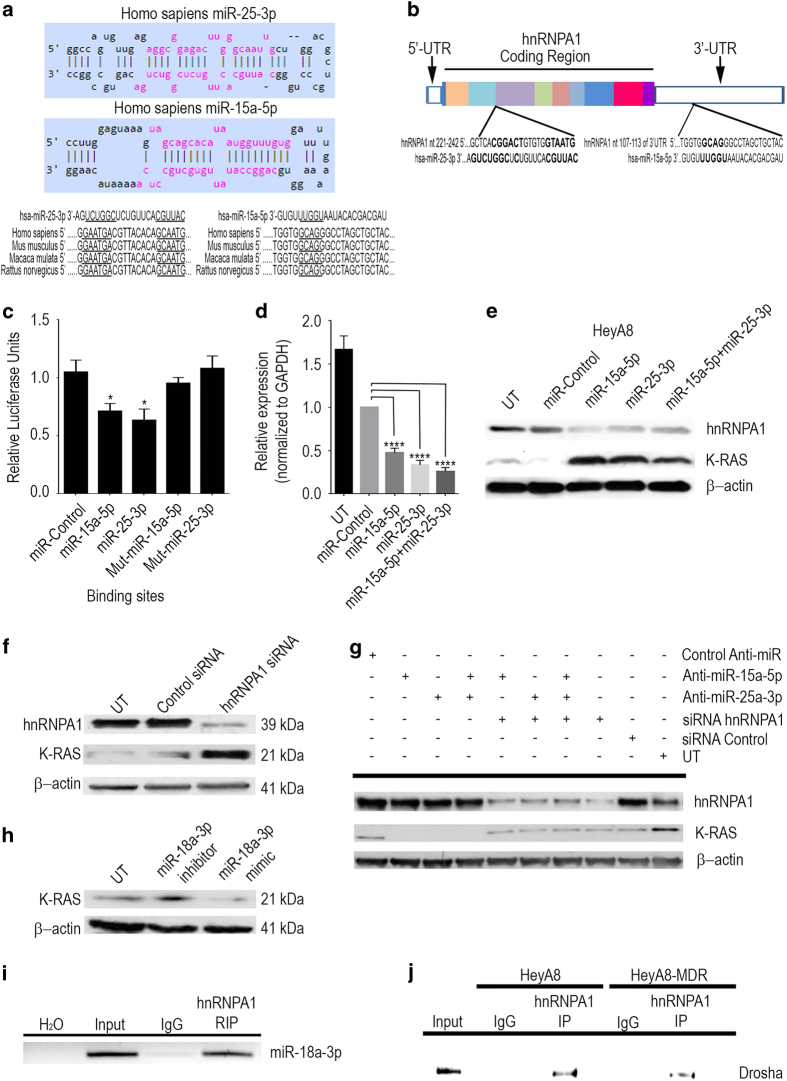
hnRNPA1 is a direct target of miR-25-3p and miR-15-5p in ovarian cancer cells. The sites for miR-25-3p and miR-15a-5p targeting of the *hnRNPA1* 3′-untranslated region (UTR) were predicted. (**a**) miR-25-3p and miR-15a-5p targeting sequences of the *hnRNPA1* 3′-UTR is evolutionarily conserved over the three species tested (*Homo sapiens*: human; *Mus musculus*: mouse; *Rattus novergicus*: rat). The targeting sites are underlined. (**b**) This schematic illustration depicts the localization of the binding sites for miR-25-3p and miR-15a-5p targeting sequences of *hnRNPA1*, each color indicate an exonic region of the gene and boxes in white represent the 5′ and 3′ UTR. UT, untreated HEK293T cells were co-transfected with wild-type or mutant reporter and the miR-25-3p and/or miR-15a-5p mimic or negative control (NC mimic). After 48 h, luciferase/Renilla activity was measured *P<0.05. (**c**) The expression of *hnRNPA1* was examined by quantitative reverse transcriptase-PCR (**d**, ****P<0.0001) and western blotting analysis (**e**) in HeyA8 cells that were co-transfected with miR-25-3p and/or miR-15a-5p mimic (or NC mimic). β-Actin was used as the endogenous control. (**f**) hnRNPA1 and K-RAS were detected by western blotting after transfection with hnRNPA1 or control siRNA. (**g**) hnRNPA1 and K-RAS were detected by western blotting after transfection with miR-25-3p and/or miR-15a-5p inhibitors and/or co-transfected with hnRNPA1 or control siRNA. (**h**) K-RAS was detected by western blotting after transfection with miR-18-3p inhibitor or mimic. (**i**) Endogenous hnRNPA1 was immunoprecipitated from total HeyA8 extracts and the pulled-down RNA was isolated and reverse-transcribed with the specific primer for miR-18a-3p PCR amplification. (**j**) Direct interaction between hnRNPA1 and Drosha was shown by pull-down assay in HeyA8 and HeyA8-MDR cells.

We first determined whether miR-15a-5p and miR-25-3p target hnRNPA1. Analysis of the seed sequences of mature miR-15a-5p and miR-25-3p showed that they were conserved among human and mouse species (Figure 2a). The putative secondary RNA hybrids of human miR-15a-5p or miR-25-3p and *hnRNPA1* mRNA is shown in Figure 2b. To determine whether *hnRNPA1* is regulated by miR-15a-5p and/or miR-25-3p, we performed a luciferase assay to examine the regulatory effects of these miRNAs on the *hnRNPA1* 3′-UTR. When the reporter gene vector was co-expressed with either of the two miRNAs, miR-25-3p expression led to a 37% reduction in reporter gene activity (*P*<0.05), while miR-15a-5p reduced reporter gene activity by 29% (Figure 2c). Disruption of the single miR-15a-5p and miR-25-3p binding site in the *hnRNPA1* 3′-UTR resulted in a loss of responsiveness to the miRNA, demonstrating that these binding sites are functionally active (Figure 2c). Next, to examine the effects of miR-15a-5p and miR-25-3p on the expression of *hnRNPA1*, HeyA8 cells were transfected with miR-15a-5p, miR-25-3p or the combination, and the levels of *hnRNPA1* mRNA were measured by quantitative reverse transcriptase-PCR. Quantities of *hnRNPA1* mRNA were 53, 67, and 75% lower, respectively, than in cells transfected with the control mimic (Figure 2d). These findings were confirmed by western blotting (Figure 2e).

We then downregulated the hnRNPA1 expression in HeyA8 cells by small interfering RNA (siRNA) and assessed the expression of K-RAS protein. Western blotting confirmed hnRNPA1 knockdown and showed that K-RAS protein expression was significantly increased (Figure 2f). We used two different siRNAs against hnRNPA1 (NM.031157) from which we selected the one that had the best silencing effect (Supplementary Figure S3a). In order to get a solid evidence that hnRNPA1 affects K-RAS expression through miR18a-3p, we performed a rescue experiment. When the HeyA8 cell were treated with either anti-miR-15a-5p or anti-miR-25a-3p, the K-RAS expression was downmodulated, but when hnRNP A1 was silenced by the treatment with siRNA, the expression of K-RAS was restored (Figure 2g). Moreover, in order to determine whether miR-18a-3p is responsible of the regulation of K-RAS, we performed western blotting to examine the regulatory effect of miRNA-18a-3p after the treatment of either the mimic or the anti-miR for miRNA-18a-3p. Although the treatment with the inhibitor of miR-18a-3p increased the expression of K-RAS, the treatment of the mimic of miR-18-3p decreased the expression of K-RAS (Figure 2h). RNA immunoprecipitation assay revealed that the miRNA-18a-3p bound to hnRNPA1 protein in HeyA8 cells (Figure 2i). We also detected a positive association between the hnRNPA1 ratio and Drosha expression in these cells (Figure 2j). Not only did we detect the binding of the hnRNPA1 protein to the miRNA processing complex of Drosha but we also observed specific binding patterns corresponding to different interactions in HeyA8 and HeyA8-MDR cells (Figure 2j). These results suggest that either miR-15a-5p or miR-25-3p can downregulate the hnRNPA1 expression and that this downregulation increases the expression of K-RAS. Moreover, the interaction between Drosha and hnRNPA1 is weaker in resistant cells, which suggests that the processing of miRNA-18a-3p in these cells is low and therefore the expression of K-RAS is increased.

### Suppression of miR-15a-5p and miR-25-3p reduces cell proliferation, migration and invasion

**Figure 3:**
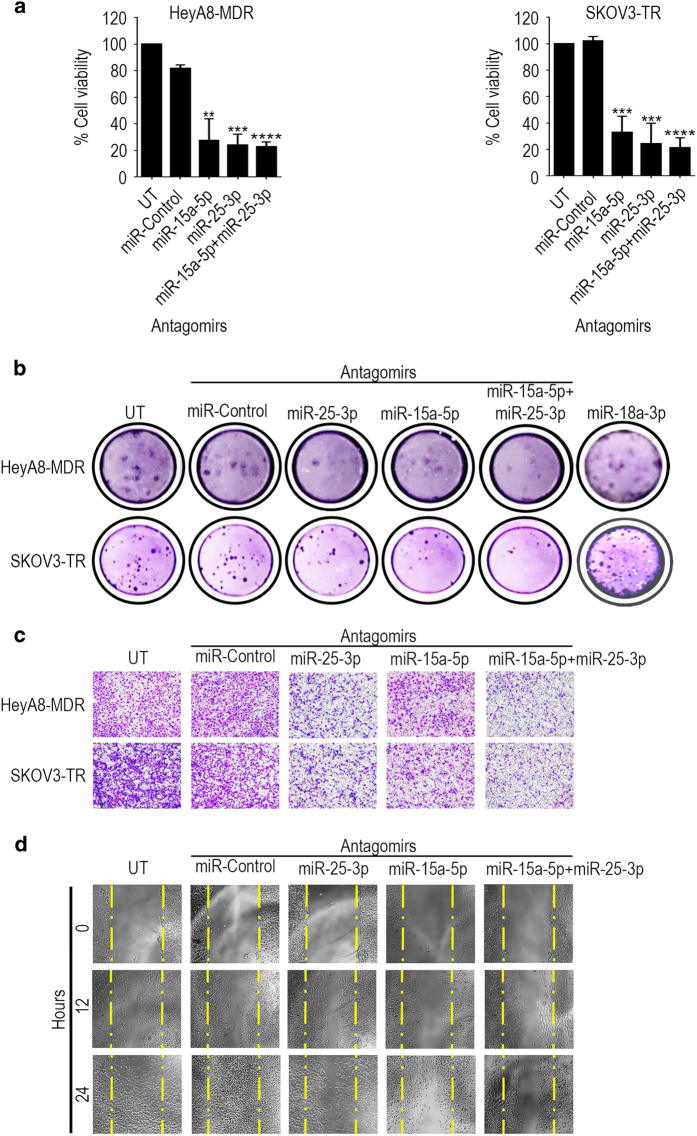
miR-25-3p and/or miR-15a-5p promote proliferation, colony formation, migration and invasion of ovarian cancer cells. (**a**) Cell viability, (**b**) colony formation and (**c**) invasion of HeyA8-MDR and SKOV3-TR cells treated with anti-miR-25-3p, anti-miR-15a-5p, a combination of the two anti-miRs or negative control anti-miR. UT, untreated. (**d**) Migration of HeyA8-MDR cells treated with anti-miR-25-3p, anti-miR-15a-5p, a combination of the two anti-miRs or negative control anti-miR. Yellow lines delimited the scratch starting point. Results for cell viability are presented as normalized means±s.d. *P<0.05; **P<0.01; ***P<0.001; ****P<0.0001.

To determine the contribution of miR-15a-5p and miR-25-3p to the aggressiveness of chemotherapy-resistant ovarian cancer cells, we carried out several cellular functional assays in resistant HeyA8-MDR and SKOV3-TR cells in which the miRNAs were inhibited. An assay using Alamar blue reagent to measure proliferation in cells treated with anti-miR-15a-5p (27.74% and 33.14% of cell viability, respectively), anti-miR-25-3p (24.13% and 24.47% of cell viability, respectively) or the combination (22.88% and 21.58% of cell viability, respectively) revealed significant reductions in proliferation induced by miRNA inhibition relative to controls and no significant difference between cells treated with either anti-miRNA (anti-miR) alone or the combination (Figure 3a). Similarly, a colony-formation assay revealed reductions in colony formation by cells treated with either anti-miR-15a-5p (6.33 and 14.33 colonies from 12.33 and 36.33 of the control, respectively) or anti-miR-25-3p (5.33 and 15.66 colonies from 12.33 and 36.33 of the control, respectively) alone, but in this case colony formation was reduced to a significantly greater extent in the combination treated (4.33 and 10.33 colonies from 12.33 and 36.33 of the control, respectively) cells (Figure 3b). We also did a rescue experiment by treating cells with anti-miR-18a-5p, which results an increase (16.33 and 33.33 colonies from 18 and 40 of the control, respectively) of the colonies (Figure 3b). Cells in which hnRNPA1 was silenced, however, showed no change in proliferation with hnRNPA1 siRNA treatment (Supplementary Figure S3b); moreover, these cells expressed greater aggressiveness as determined by a multiplex assay showing increases in pro-invasive factors such as matrix metalloproteinases (MMPs) MMP-1 and MMP-9 and in pro-angiogenic factors such as fibroblast growth factor (FGF)-2, vascular endothelial growth factor (VEGF)-D and VEGF-C, which have important roles in tumor-mediated migration, invasion and angiogenesis (Supplementary Figure S3c and d). Assessment of the migration and invasion potential of HeyA8-MDR and SKOV3-TR cells treated with anti-miR-15a-5p, anti-miR-25-3p or the combination was consistent with these findings: migration and invasiveness of cells treated with either anti-miR alone were reduced compared with controls, and this effect was even more potent in cells treated with both anti-miRs (Figure 3c and d).

### Silencing of miR-15a-5p and miR-25-3p has antitumor effects in a murine model of chemoresistant ovarian cancer

**Figure 4:**
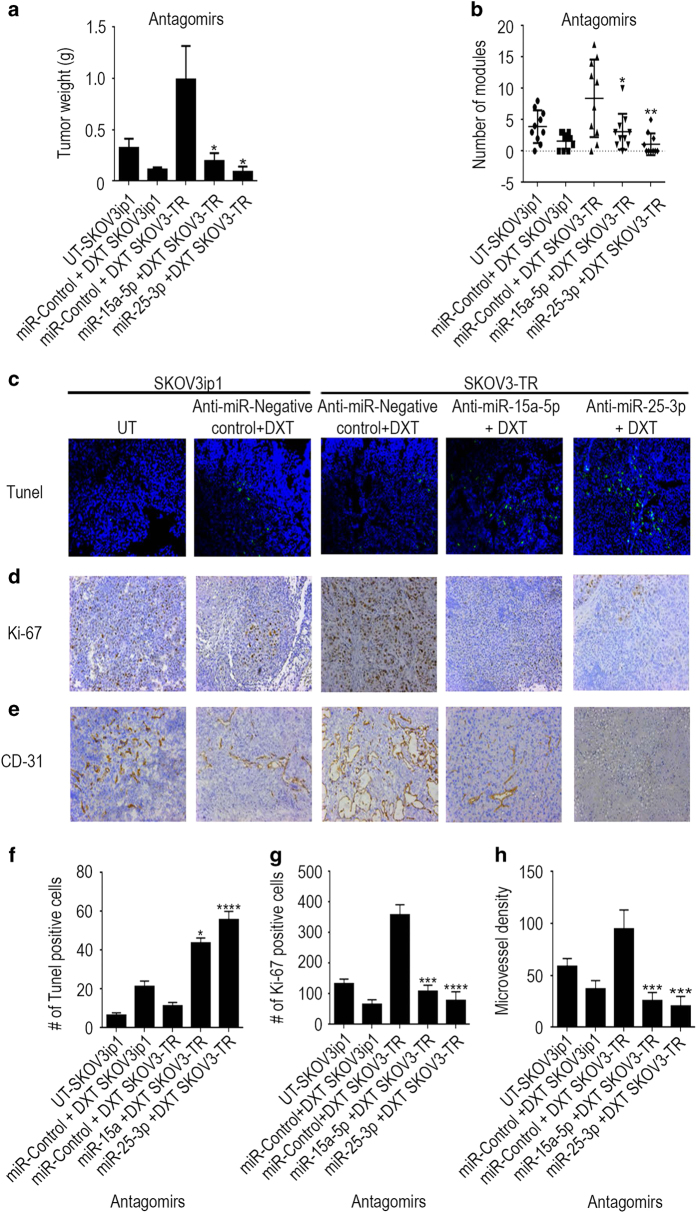
Anti-miR-25-3p, anti-miR-15a-5p and docetaxel (DXT) have antitumor effects in ovarian cancer mouse models. SKOV3IP1 or resistant SKOV3-TR ovarian tumor-bearing mice treated with anti-miR-25-3p and/or anti-miR-15a-5p (200 μg kg^−1^ body weight/intravenous) plus DXT (75 μg intraperitoneally) for 5 weeks exhibited lower tumor weights (**a**), fewer tumor nodules (**b**), more TUNEL (terminal deoxinucleotidyl transferase-mediated dUTP-fluorescein nick end labeling)-positive cells (**c**), lower Ki67 index (**d**) and lower microvessel density (CD31-positive staining) (**e**) than tumor-bearing mice treated with negative control (NC) anti-miR plus DXT. Quantification of apoptosis (**f**), proliferation (**g**) and angiogenesis (**h**) and *in vivo*. Data are presented as means±s.d. *P<0.05; **P<0.01; ***P<0.001; ****P<0.0001; *n*=10 mice per group.

Next, we evaluated the effects of miR-15a-5p and miR-25-3p silencing on tumor growth and metastasis *in vivo* in two murine orthotopic models of ovarian cancer established by intraperitoneal injection of parental SKOV3IP1 or resistant SKOV3-TR cells. Tumor-bearing mice were treated twice a week with anti-miR-15a-5p or anti-miR-25-3p (or a negative control anti-miR) delivered via a 1,2-dioleoyl-sn-glycero-3-phosphatidylcholine (DOPC) liposomal platform that we developed previously [23] plus docetaxel (DXT), a chemotherapeutic agent often used to treat ovarian cancer. Upon analysis of tumor growth and metastasis at the end of the experiment, we observed significant reductions from controls in gross tumor weight in the mice treated with one of the two anti-miRs plus DXT (anti-miR-15a-5p+DXT=0.191 g, anti-miR-25-3p+ DXT=0.086 g), both the negative control SKOV3IP1 (negative control+DXT=0.0112 g) tumor-bearing mice and the SKOV3-TR (negative control+DXT=0.986 g) tumor-bearing mice (Figure 4a). The largest tumor weight reduction was observed in the groups treated with anti-miR-25-3p plus DXT (0.0865 g) (Figure 4a). Similarly, the greatest reductions in total numbers of tumor nodules were in mice treated with the anti-miR-25-3p plus DXT combination compared with the negative control anti-miR plus DXT (Figure 4b).

Immunofluorescence staining of tumor tissues from all five groups subjected to TUNEL (terminal deoxinucleotidyl transferase-mediated dUTP-fluorescein nick end labeling) assay showed significant increases in apoptosis in the negative control anti-miR plus DXT-treated SKOV3IP1 mice and the anti-miR-15a-5p or anti-miR-25-3p plus DXT-treated SKOV3-TR mice compared with the control group (Figure 4c and f). Quantitation of proliferation via the Ki67 index showed significant reductions in cell proliferation in the SKOV3IP1 mice treated with the negative control anti-miR plus DXT and the SKOV3-TR mice treated with anti-miR-15a-5p or anti-miR-25-3p plus DXT compared with the control group (Figure 4d and g). To support the *in vitro* observations, we also evaluated the effect of miR-15a-5p and miR-25-3p silencing on angiogenesis in this ovarian cancer model. Microvessel density was reduced in the SKOV3IP1 mice treated with negative control anti-miR plus DXT and in the SKOV3-TR mice treated with anti-miR-15a-5p or anti-miR-25-3p plus DXT compared with the control group (Figure 4e and h). Together, these results indicate that anti-miR-15a-5p or anti-miR-25-3p combined with DXT exerted robust antitumor effects, indicating the clinical potential of this regimen.

### Deregulation of K-RAS, miR-18a-3p, miR-15a-5p and miR-25-3p in ovarian cancer

**Figure 5:**
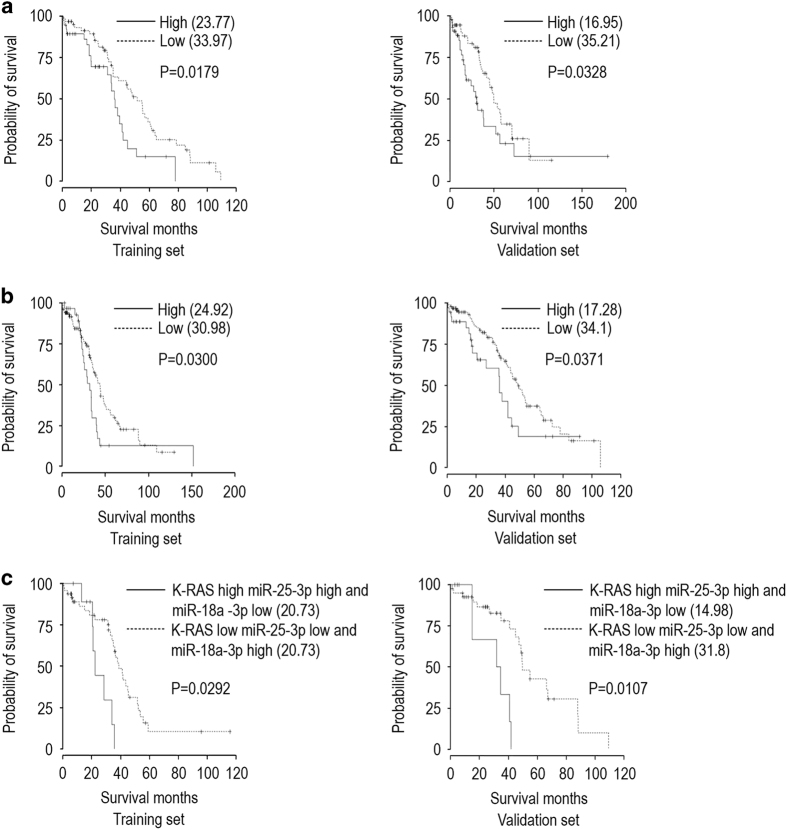
Patterns of miR-25-3p, miR-15-5p, miR 18 a-3p and K-RAS expression predict overall survival (OS) in the The Cancer Genome Atlas (TCGA) ovarian cancer patient data set. (**a**) Kaplan–Meier survival curves for patients from the TCGA ovarian cancer database as a function of the miR-15a-5p (cutoff=0.66) and K-RAS expression of their tumor (training set; cutoff=0.41, *P*=0.0179, validation set; *P*=0.0328). (**b**) Kaplan–Meier survival curves for the same patients as a function of the miR-25-3p (cutoff=0.66) and K-RAS expression of their tumor (training set; cutoff=0.41, *P*=0.0300, validation set; *P*=0.0371). (**c**) Kaplan–Meier survival curves for the same patients as a function of the miR-25-3p (cutoff=0.63), miR-18a-3p (cutoff=0.47), and K-RAS expression of their tumor (training set; cutoff=0.66, *P*=0.0292, validation set *P*=0.0107). OS (defined as interval in months from the date of initial surgical resection to the date of death or last follow-up) for patients (Training and Validation sets) with high-grade serous ovarian adenocarcinoma classified according to the expression of K-RAS, miR-25-3p, miR-18a-3p and miR-15a-5p (AGILENT and AFFYMETRIX Microarray Platforms).

We next investigated the expression patterns of K-RAS, miR-18a-3p, miR-15a-5p and miR-25-3p in ovarian tumors by analyzing the publicly available ovarian cancer database of The Cancer Genome Atlas (TCGA; http://cancergenome.nih.gov/). We then performed Kaplan–Meier survival analyses of the patients in that database grouped by the levels of expression of K-RAS and miR-18a-3p in their tumor tissue. We observed significantly longer overall survival (OS) in patients whose tumors expressed a low level of K-RAS and a high level of miR-18a-3p than in those whose tumors expressed a high level of K-RAS and a low level of miR-18a-3p (median OS (months), training set: high K-RAS/low miR=30.89, low K-RAS/high miR=34.97; validation set: high K-RAS/low miR=27.54, low K-RAS/high miR=31.65; training set, *P*=0.0323; validation set, *P*=0.0120; Supplementary Figure S4a). A further analysis of the associations between K-RAS and miR-15a-5p revealed significantly longer OS in patients whose tumors expressed low levels of K-RAS and miR-15-5p than in those whose tumors expressed high levels of K-RAS and miR-15-5p (median OS (months), training set: high K-RAS/miR=23.77, low K-RAS/miR=33.97; validation set, high K-RAS/miR=19.95, low K-RAS/miR=35.21; training set, *P*=0.0179; validation set, *P*=0.0328; Figure 5a). Moreover, associations between K-RAS and miR-25-3p revealed significantly longer OS in patients whose tumors expressed low levels of K-RAS and miR-25-3p than in those whose tumors expressed high levels of K-RAS and miR-25-3p (median OS (months), training set: high K-RAS/miR=24.92, low K-RAS/miR=30.98; validation set: high K-RAS/miR=17.28, low K-RAS/miR=34.1; training set, *P*=0.0300; validation set, *P*=0.0371; Figure 5b). Our comparison of OS effects due to expression of a single miRNA and K-RAS in ovarian tumor samples identified a direct associations among high levels of K-RAS and miR-25-3p and low expression levels of miR-18a-3p or between low levels of expression of K-RAS and miR-25-3p and high levels of miR-18a-3p in these tumors. OS was significantly longer for patients whose tumor expressed low levels of K-RAS and miR-25-3p and a high level of miR-18a-3p than for those whose tumor expressed high levels of K-RAS and miR-25-3p and a low level of miR-18a-3p (median OS (months), training set: high K-RAS=20.73, low K-RAS=31.49; validation set: high K-RAS=14.98, low K-RAS=31.8; training set, *P*=0.0292; validation set, *P*=0.0107; Figure 5c). These data proved the fact that regulation of hnRNPA1 by miRNAs controls the miR-18a–*K-RAS* axis in chemotherapy-resistant ovarian cancer.

## Discussion

**Figure 6:**
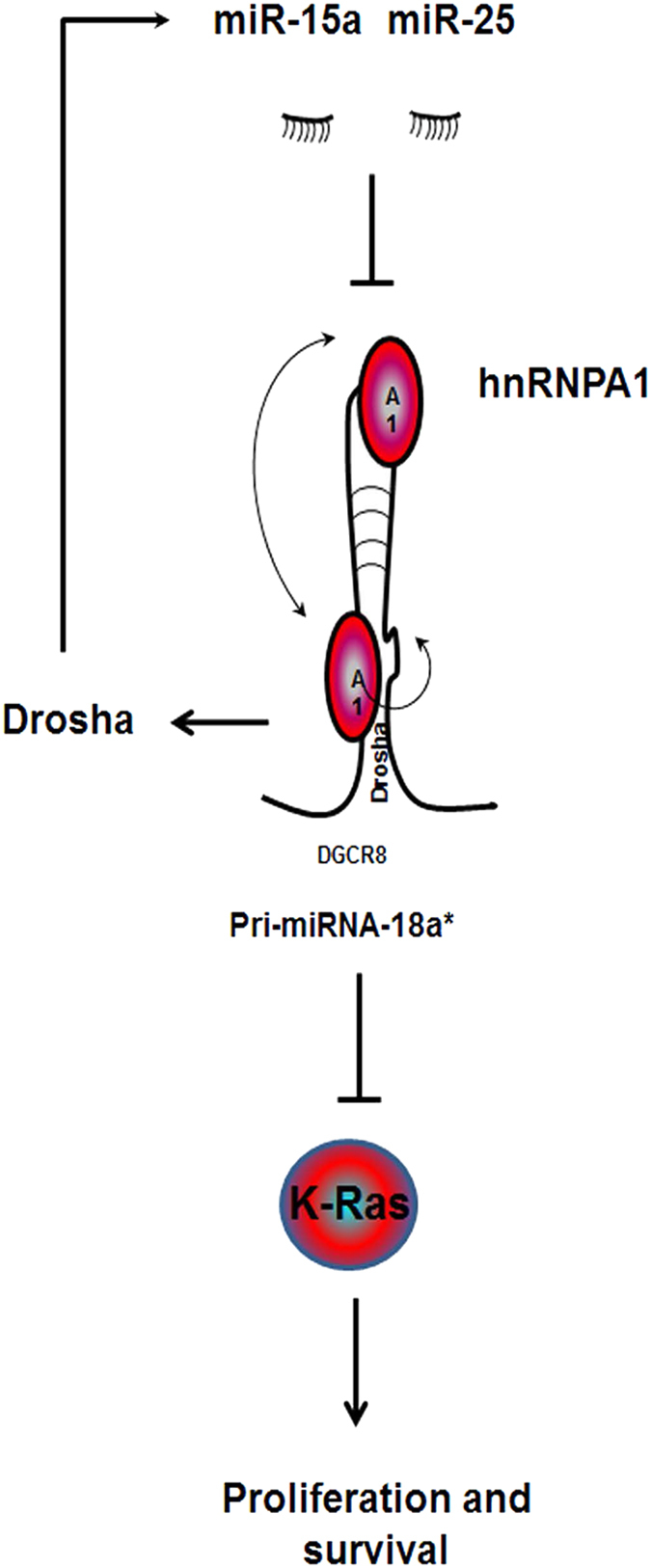
Schematic illustration shows targeting of the hnRNPA1/pri-miRNA-18*/K-RAS pathway by miR-25-3p and miR-15a-5p.

We elucidated a novel mechanism of miRNA self-regulatory activity whereby the oncogenic expression of the RNA-binding protein hnRNPA1 is downregulated by miR-25-3p and/or miR-15a-5p, which causes hnRNPA1 (a facilitator of miR-18a-3p) in turn to downregulate the expression of tumor-suppressor miR-18a-3p, thus leading to an increase in the expression of K-RAS required for increasing survival in ovarian cancer chemotherapy-resistant cells (Figure 6). Reversing this process by targeting the oncogenic miRNAs (miR-25-3p and/or miR-15a-5p) and modulating the ratio between the tumor-suppressor miRNA (miR-18a-3p) and its downstream target (KRAS) has direct effect on proliferation and survival.

The combined effect between DXT and the silencing of miR-25-3p and/or miR-15a-5p and the subsequent inhibition of K-RAS led to a synergistic enhancement in response than either effect alone by minimizing the opportunity for tumor cells to survive and develop resistance.

Our own work reported here and that of others show that miR-25-3p and miR-15-5p are involved in chemoresistance [24–27], and hnRNP signaling in taxane resistance has been described previously [28]. Hence, hnRNPA1 pathway inhibition (via diverse mechanisms) might be a necessary event for the development of DXT resistance. Published work has suggested that inhibition of miR-25-3p might be effective in the treatment of ovarian cancer [29], but this inhibition has not been associated with the miR-25-3p and/or miR-15a-5p-hnRNPA1 loop. Our findings reported suggest that inhibition of hnRNPA1-miRNA interactions is key to overcoming acquired resistance to DXT in ovarian cancer cells.

Our study has identified the dependence of miR-25-3p- and/or miR-15a-5p-regulated chemoresistance in ovarian cancer on miR-18a-3p-K-RAS signaling and its underlying mechanisms (see Figure 6 for model). These data suggests an alternative therapeutic approach for patients whose tumor expresses high levels of K-RAS and miR-25-3p or miR-15a-5p. The upfront polytherapeutic strategy employing a combination of existing therapies (for example, DXT) and anti-miRs to miR-25-3p and miR-15a-5p supported by our findings might convert the incomplete and temporary responses obtained with DXT (Figure 4), and could be applicable to potent chemotherapeutics agents such as cisplatin and abraxane, into a complete and sustained remission in ovarian cancer patients.

## Materials and methods

### Reagents

DXT was purchased from Aventis (Bridgewater, NJ, USA); restriction enzymes were obtained from New England BioLabs (Ipswich, MA, USA). siRNA, Tert-butanol and β-actin monoclonal antibody and mouse and rabbit horseradish peroxidase-conjugated secondary antibodies were purchased from Sigma (St Louis, MO, USA). Anti-miRNA were purchased from Ambion Thermo Fisher Scientific Inc. (Waltham, MA, USA) DOPC was purchased from Avanti Polar Lipids (Alabaster, AL, USA).

### Cell line maintenance and siRNA and miRNA transfections

All cell lines were maintained in 5% CO_2_ at 37 °C. Ovarian cancer (SKOV3IP1, HeyA8 and A2780) cells were obtained from the American Type Culture Collection (Manassas, VA, USA). Chemotherapy-resistant derivatives SKOV3-TR, HeyA8-MDR and A2780-CP20 were developed as described by Vivas-mejia *et al.* [30]. SKOV3-TR cells were obtained by clonal selection and maintained in RPMI 1640 supplemented with 10% fetal bovine serum (FBS) and 150 ng ml^−1^ paclitaxel. HeyA8-MDR cells were obtained by clonal selection and maintained in RPMI 1640 supplemented with 10% FBS and 300 ng ml^−1^ paclitaxel. All the others cell lines were maintained in RPMI-1640 medium supplemented with 10–15% FBS and 0.1% gentamicin sulfate (Gemini Bioproducts, Calabasas, CA, USA). All cell lines were routinely tested to confirm the absence of mycoplasma, and all *in vitro* experiments were conducted with 60–80% confluent cultures. All siRNA and miRNA transfections were performed with RNAiMAX (Invitrogen, Carlsbad, CA, USA) reagent using the forward transfection protocol from the manufacturer. Medium was changed 4 h after transfection to minimize toxicity.

### In vivo models

Female athymic nude mice were purchased from Taconic Farms (Hudson, NY, USA). These animals were cared for according to guidelines set forth by the American Association for Accreditation of Laboratory Animal Care and the US Public Health Service policy on Humane Care and Use of Laboratory Animals. All mouse studies were approved and supervised by The University of Texas MD Anderson Cancer Center Institutional Animal Care and Use Committee. All animals (*N*=60 mice) were aged 6–8 weeks at the time of injection.

Orthotopic models of ovarian cancer were developed as described previously [30]. For all animal experiments, SKOV3IP1 or SKOV3-TR cells were harvested using trypsin-EDTA, neutralized with FBS-containing medium, washed and resuspended in appropriate cell numbers in Hanks balanced salt solution (Gibco, Carlsbad, CA, USA) prior to intraperitoneal injection. Mice were randomly assigned to receive an anti-miR incorporated into neutral DOPC nanoliposomes alone or in combination with DXT. For all therapeutic experiments, the dose of anti-miRNA was 200 μg kg^−1^, as described previously [6, 23, 31]. Anti-miRs were administered via intravenously injection twice weekly beginning 1 week after cell injection and continued for approximately 5 weeks. DXT (75 μg per injection) was administered weekly. In all experiments, mice in any group were killed when they became moribund by the cervical dislocation method. They were subjected to necropsy after death and tumors were harvested. Tumor weight and the number and location of tumor nodules were recorded. Tumor tissue was fixed in formalin for paraffin embedding, frozen in optimal cutting temperature medium to prepare frozen slides or snap-frozen for lysate preparation.

### Liposomal nanoparticle preparation

Anti-miR for *in vivo* intratumoral delivery was incorporated into DOPC liposomes, as previously described [23]. DOPC and anti-miR were mixed in the presence of excess tertiary butanol at a ratio of 1:10 w/w) anti-miR. Tween-20 was added to the mixture in a ratio of 1:19. The mixture was subjected to vortex, to freeze in an acetone/dry ice bath and to lyophilization. Before *in vivo* administration, this preparation was hydrated with phosphate-buffered saline solution at room temperature at a concentration of 200 μg anti-miR per kg per injection.

### TCGA data and bioinformatic analysis

Agilent 44K gene expression and Agilent human miRNA microarray 8×15K miRNA expression data and clinical information were obtained from the open access and controlled-access tiers of the TCGA data portal, with NIH approval. Alignment of sample identifiers yielded 456 tumor cases with all information available at the time of data retrieval from the TCGA. Pearson correlation test, Kaplan–Meier estimate and Mantel–Cox survival analyses were performed using the R software version 2.10.0. Significance was defined as *P*<0.05.

### Target gene-binding sites and luciferase reporter assays for the hnRNPA1 3′-UTR

The putative miRNA binding sites on the *hnRNPA1* 3′-UTR were predicted via bioinformatics analysis using several algorithms for predicting miRNA targets available from the following public sites: http://www.microrna.org (miRanda algorithm); http://www.targetscan.org (TargetScan algorithm); http://genie.weizmann.ac.il/pubs/mir07 (PITA algorithm); http://cbcsrv.watson.ibm.com (RNA22 algorithm); http://diana.cslab.ece.ntua.gr/microT (microT algorithm); and http://genome.ucsc.edu/cgi-bin/hgTables?command=start together with http://pictar.mdc-berlin.de/ (PicTar algorithm). The miRNAs predicted by this analysis to target hnRNPA1 were further shortlisted by validation with real-time PCR. The pGL3 Luciferase Reporter Vectors for the predicted binding sites of the 3′-UTR region of *hnRNPA1* were obtained from Promega (Madison, WI, USA).

HEK239 cells were transfected with lipofectamine 2000 reagent in a 96-well plate with scrambled control or an miR-25-3p or miR-15a-5p mimic (100 nM; Ambion, Austin, TX, USA) along with the 3′-UTR reporter gene and Renilla luciferase vector control construct (phRL-TK). After 24 h of transfection, luciferase activity was determined with the Dual-Luciferase Reporter Assay System Kit using a microplate luminometer per the manufacturer’s guidelines (Biotek, Winooski, VT, USA). Luciferase activity was normalized against the phRL-TK control construct, and an empty luciferase reporter vector was used as a negative control. The ratios obtained were further normalized according to the scrambled control.

### Immunoblotting

Lysates from cultured cells were prepared in modified RIPA buffer (50 mM Tris-HCl (pH 7.4), 150 mM NaCl, 1% Triton, 0.5% deoxycholate) plus 25 μg ml^−1^ leupeptin, 10 μg ml^−1^ aprotinin, 2 mM EDTA and 1 mM sodium orthovandate. The protein concentrations were determined by using a BCA Protein Assay Reagent kit (Pierce Biotechnology, Rockford, IL, USA). Lysates were loaded and separated on sodium dodecyl sulfate-polyacrylamide gels. Proteins were transferred to a nitrocellulose membrane by wet electrophoresis (Bio-Rad Laboratories, Hercules, CA, USA) overnight, blocked with 5% bovine serum albumin for 30 min and then incubated at 4 °C overnight with primary antibody (hnRNPA1, K-RAS; Millipore, Darmstadt, Germany). After washing with Tris-buffered saline solution containing Tween 20, the membranes were incubated with horseradish peroxidase-conjugated horse anti-mouse or rabbit IgG (1:1000; Cell Signaling Technology, Danvers, MA, USA) for 1 h. Horseradish peroxidase was visualized using an enhanced chemiluminescence detection kit (GE Healthcare, Wauwatosa, WI, USA). To confirm equal sample loading, the blots were probed with an antibody specific for β-actin (0.1 μg ml^−1^; Sigma, St Louis, MO, USA).

### Quantitative real-time PCR

For mRNA quantification, total RNA was isolated using the RNeasy Kit (Qiagen, Valencia, CA, USA). cDNA was synthesized from 1000 ng of RNA by using a Verso cDNA Kit (Thermo Scientific, Waltham, MA, USA) per the manufacturer’s instructions. mRNA levels were analyzed on a 7500 Fast Real-Time PCR System (Applied Biosystems, Foster City, CA, USA) with SYBR green-based real-time PCR for all genes except the one specified. Dicer and Drosha Taqman assays (Life Technologies, Carlsbad, CA, USA) were performed. Reverse-transcribed RNA and 100 ng μl^−1^ each of sense and antisense primers in a total volume of 20 μl were subjected to semiquantitative real-time PCR.

For miRNA quantification, total RNA was isolated using the Trizol/isopropanol total RNA precipitation method (Invitrogen). For quantification of pri-miRNA and mature miRNA, Taqman miRNA assays (Life Technologies) were used and real-time PCR was carried out according to the manufacturer’s instructions. *RNU6B* (for mature miRNAs) or *18S* (for pri-miRNA and precursor miRNAs) were used as housekeeping genes.

### Migration and invasion assays

Modified Boyden chambers (Coster, Boston, MA, USA) coated with 0.1% gelatin (migration) or extracellular matrix components (invasion) were used. HeyA8-MDR and SKOV3-TR cells (1×10^5^) suspended in 100 μl of serum-free medium were added into the upper chambers 24 h after miRNA/siRNA transfection. Complete cell medium containing 10% FBS (500 μl) was added to the bottom chamber as a chemo-attractant. The chambers were incubated at 37 °C in 5% CO_2_ for 6 h (migration) or overnight (invasion). After incubation, the cells in the upper chambers were removed with cotton swabs. Cells that had migrated or invaded the intervening coating layer were fixed and stained and counted by light microscopy. Cells from five random fields were counted. Experiments were carried out in duplicate and performed three times.

### Inmunoprecipitation and RIP assay

HeyA8 and HeyA8-MDR cells were subjected to lysis and incubated with hnRNPA1 antibody. After incubation, the lysates were subjected to an hnRNPA1 Pull-down and Detection Kit (Thermo Scientific) according to the manufacturer’s instructions.

For the RNA immunoprecipitation assay, we used the Magna RIP Kit (Millipore) according to the manufacturer’s instructions. Cells were prepared in RIP lysis buffer, and the RNA-protein complexes were immunoprecipitated with magnetic beads using anti-hnRNPA1 or normal mouse IgG (control). Co-purified RNA was extracted using phenol:chloroform:isoamyl alcohol and subjected to reverse transcription and agarose electrophoretic running analysis.

### Measurement of pro-angiogenic and pro-invasive factors

Levels of pro-angiogenic factors angiopoietin-2, bone morphogenetic protein-9, epidermal growth factor, endoglin, endothelin-1, FGF-1, FGF-2, follistatin, granulocyte-colony-stimulating factor, heparin-binding epidermal growth factor, hepatocyte growth factor, interleukin-8, leptin, placenta growth factor, VEGF-A, VEGF-C and VEGF-D and pro-invasive MMPs MMP-1, MMP-2, MMP-7, MMP-9 and MMP-10 were detected in the supernatants of HeyA8-MDR cells treated with hnRNPA1 siRNA by multiplex bead immunoassay using a Luminex Kit (Millipore).

### Clonogenic assay

Briefly, 800 cells were mixed gently and plated on a 24-well plate. After incubation for 24 h, the cells were transfected with control miRNA or anti-miR-25-3p. About 2 weeks later, the cells were washed with phosphate-buffered saline and stained with crystal violet. Colonies with a diameter of >50 cells were counted. The experiment was repeated three times.

### mRNA microarray profiling

Microarray analyses of HeyA8 and HeyA8-MDR ovarian cancer cell lines were performed by using GeneChip Human U133 plus 2.0 arrays (Affymetrix, Santa Clara, CA, USA, PN 900467), containing probe sets for >47 000 characterized genes and expressed sequence tags. Sample labeling and processing, GeneChip hybridization and scanning were performed according to Affymetrix protocols. Briefly, double-stranded cDNA was synthesized from total RNA with GeneChip 3′ IVT Plus Kit (Affymetrix, PN 902416) with a T7 RNA polymerase promoter site added to its 3′ end (Genset, La Jolla, CA, USA). Biotinylated cRNAs were generated from cDNAs *in vitro* transcription and amplified by using T7 RNA polymerase. After purification of cRNAs was performed by Agencourt AMPure beads, 20 μg of cRNA was fragmented at 94 °C for 35 min. Approximately 12.5 μg of fragmented cRNA was used in a 250-μl hybridization mixture containing herring-sperm DNA (0.1 mg ml^−1^; Promega) plus bacterial and phage cRNA controls (1.5 pM BioB, 5 pM BioC, 25 pM BioD and 100 pM Cre) to serve as internal controls for hybridization efficiency. Aliquots (200 μl) of the mixture were hybridized to arrays for 16 h at 45 °C in a GeneChip Hybridization Oven 640 (Affymetrix). Each array was washed and stained with streptavidin–phycoerythrin (Invitrogen, Carlsbad, CA, USA) and amplified with biotinylated anti-streptavidin antibody (Vector Laboratories, Burlingame, CA, USA) on the GeneChip Fluidics Station 450 (Affymetrix). Arrays were scanned with the GeneArray G7 scanner (Affymetrix) to obtain image and signal intensities.
