# Author Correction: Regulation of hnRNPA1 by microRNAs controls the miR-18a-*K*-*RAS* axis in chemotherapy-resistant ovarian cancer

**DOI:** 10.1038/s41421-023-00611-6

**Published:** 2023-11-08

**Authors:** Cristian Rodriguez-Aguayo, Paloma del C Monroig, Roxana S. Redis, Emine Bayraktar, Maria I. Almeida, Cristina Ivan, Enrique Fuentes-Mattei, Mohammed H. Rashed, Arturo Chavez-Reyes, Bulent Ozpolat, Rahul Mitra, Anil K. Sood, George A. Calin, Gabriel Lopez-Berestein

**Affiliations:** 1 Department of Experimental Therapeutics, The University of Texas MD Anderson Cancer Center, Houston, TX USA; 2 Center for RNA Interference and Non-Coding RNA, The University of Texas MD Anderson Cancer Center, Houston, TX USA; 3 Instituto de Investigação e Inovação em Saúde/Institute for Research and Innovation in Health (I3S) and Instituto de Engenharia Biomédica (INEB), University of Porto, Porto, Portugal; 4 Department of Pharmacology and Toxicology, Faculty of Pharmacy, The University of Al-Azhar, Cairo, Egypt; 5 Center for Research and Advanced Studies, National Polytechnic Institute (CINVESTAV del IPN), Monterrey, Mexico; 6 Department of Gynecologic Oncology, The University of Texas MD Anderson Cancer Center, Houston, TX USA; 7 Department of Cancer Biology, The University of Texas MD Anderson Cancer Center, Houston, TX USA

Correction to: *Cell Discovery* (2017) 3, 17029

10.1038/celldisc.2017.29 published online 12 September 2017

In the original publication of this article^1^, Figure 3b, upper panel, the image of colony formation/untreated (UT) HeyA8-MDR cells group was inadvertently duplicated in the next position for the group of “miR-18a-3p treated HeyA8-MDR” during preparation. This Figure panel was an unintended error and happened when assembling the original data, but did not influence the figure legend, or the interpretation of results. We provided a corrected version of Fig. 3b colony formation for HeyA8-MDR cells with a new representative image. The correct Fig. 3b upper panel is displayed below.

In Fig. 4c, the TUNEL staining panel for the “Anti-miR-Negative control + DXT” SKOV3ip1 group was inadvertently duplicated in the next position for the SKOV3-TR group of “Anti-miR-Negative control + DXT”. We have attached a corrected version of Fig. 4c TUNEL staining panels with a new representative image for the SKOV3ip1 group of “Anti-miR-Negative control + DXT”.

The correct figure legends are below.

**Fig. 3** miR-25-3p and/or miR-15a-5p promote proliferation, colony formation, migration, and invasion of ovarian cancer cells. (**a**) Cell viability, (**b**) colony formation, and (**c**) invasion of HeyA8-MDR and SKOV3-TR cells treated with anti-miR-25-3p, anti-miR-15a-5p, a combination of the two anti-miRs or negative control anti-miR. UT, untreated. **d** Migration of HeyA8-MDR cells treated with anti-miR-25-3p, anti-miR-15a-5p, a combination of the two anti-miRs or negative control anti-miR. Yellow lines delimited the scratch starting point. Results for cell viability are presented as normalized means ± s.d. *P < 0.05; **P < 0.01; ***P < 0.001; ****P < 0.0001.
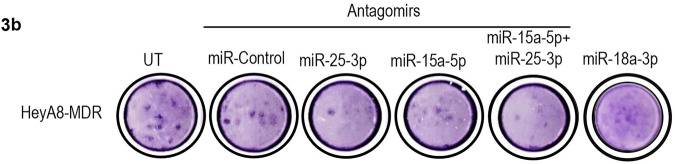


**Fig. 4** Anti-miR-25-3p, anti-miR-15a-5p, and docetaxel (DXT) have antitumor effects in ovarian cancer mouse models. SKOV3IP1 or resistant SKOV3-TR ovarian tumor-bearing mice treated with anti-miR-25-3p and/or anti-miR-15a-5p (200 μg kg^−1^ body weight/intravenous) plus DXT (75 μg intraperitoneally) for 5 weeks exhibited lower tumor weights (**a**), fewer tumor nodules (**b**), more TUNEL (terminal Deoxynucleotidyl transferase-mediated dUTP-fluorescein nick end labeling)-positive cells (**c**), lower Ki67 index (**d**), and lower microvessel density (CD31-positive staining) (**e**) than tumor-bearing mice treated with negative control (NC) anti-miR plus DXT. Quantification of apoptosis (**f**), proliferation (**g**), and angiogenesis (**h**) and in vivo. Data are presented as means ± s.d. *P < 0.05; **P < 0.01; ***P < 0.001; ****P < 0.0001; *n* = 10 mice per group.
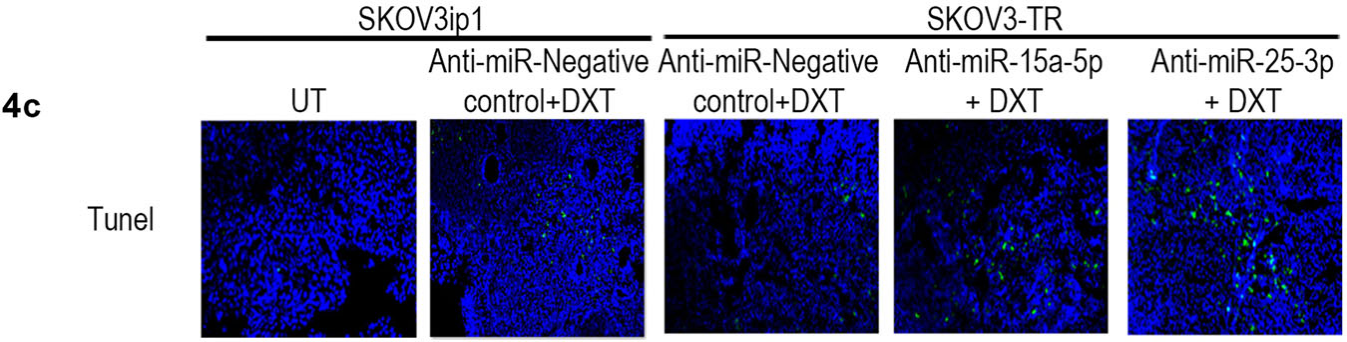


## References

[1] Rodriguez-Aguayo C (2017). Regulation of hnRNPA1 by microRNAs controls the miR-18a-K-RAS axis in chemotherapy-resistant ovarian cancer. Cell Discov..

