# Supplementary figures and images for: Regulation of hnRNPA1 by microRNAs controls the miR-18a–K-RAS axis in chemotherapy-resistant ovarian cancer
Source: Cell Discov. 2017 Sep 12;3:17029–. doi: 10.1038/celldisc.2017.29 (PMC5594916; doi:10.1038/celldisc.2017.29)

## Supplementary Figure S1

a

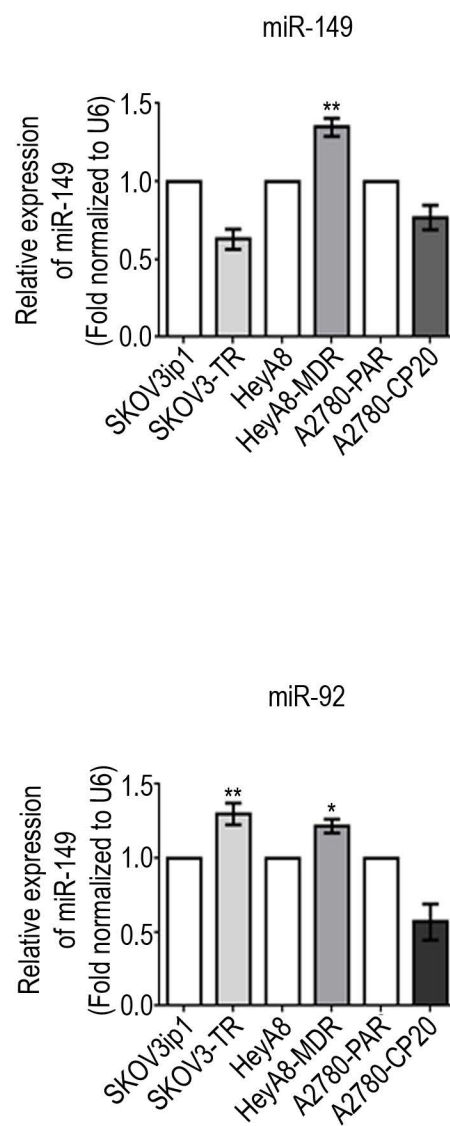

Supplement: Supplementary Figure S1 [file celldisc201729-s1.pdf]

Supplementary Figure S2

a

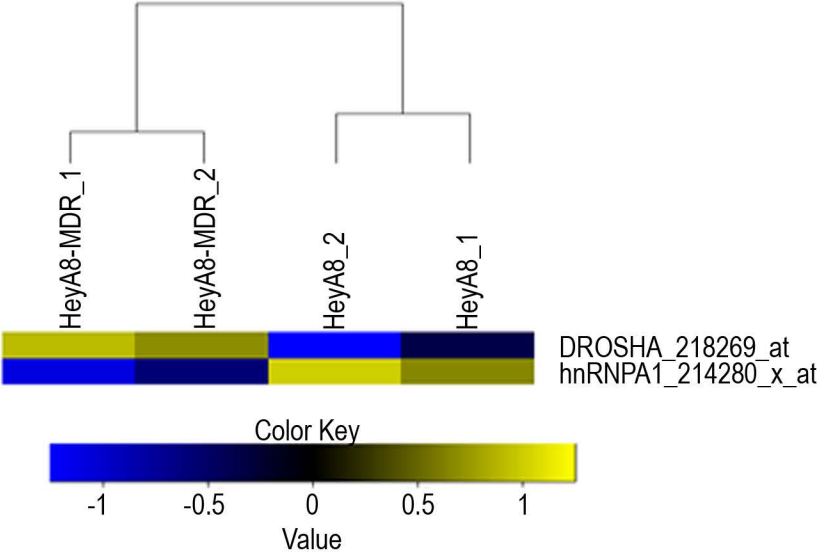

b

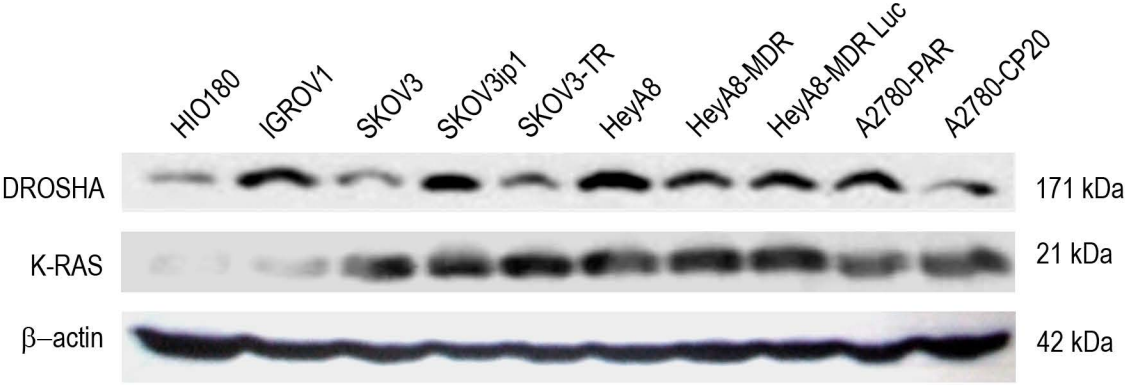

c

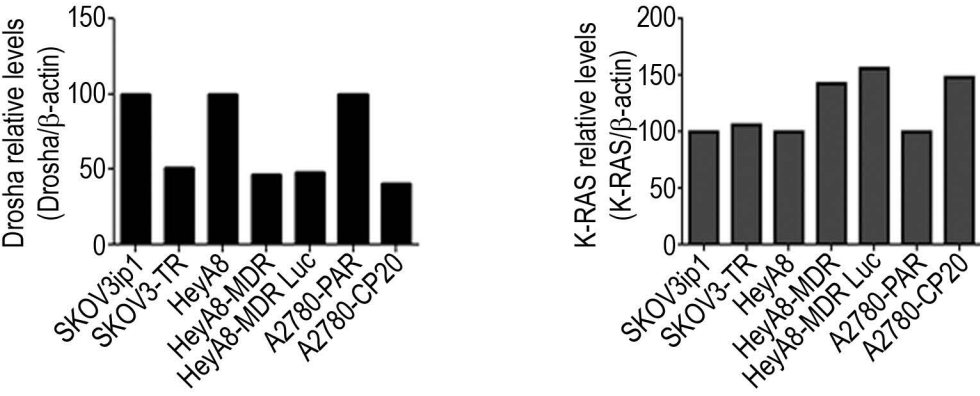

Supplement: Supplementary Figure S2 [file celldisc201729-s2.pdf]

## Supplementary Figure S3

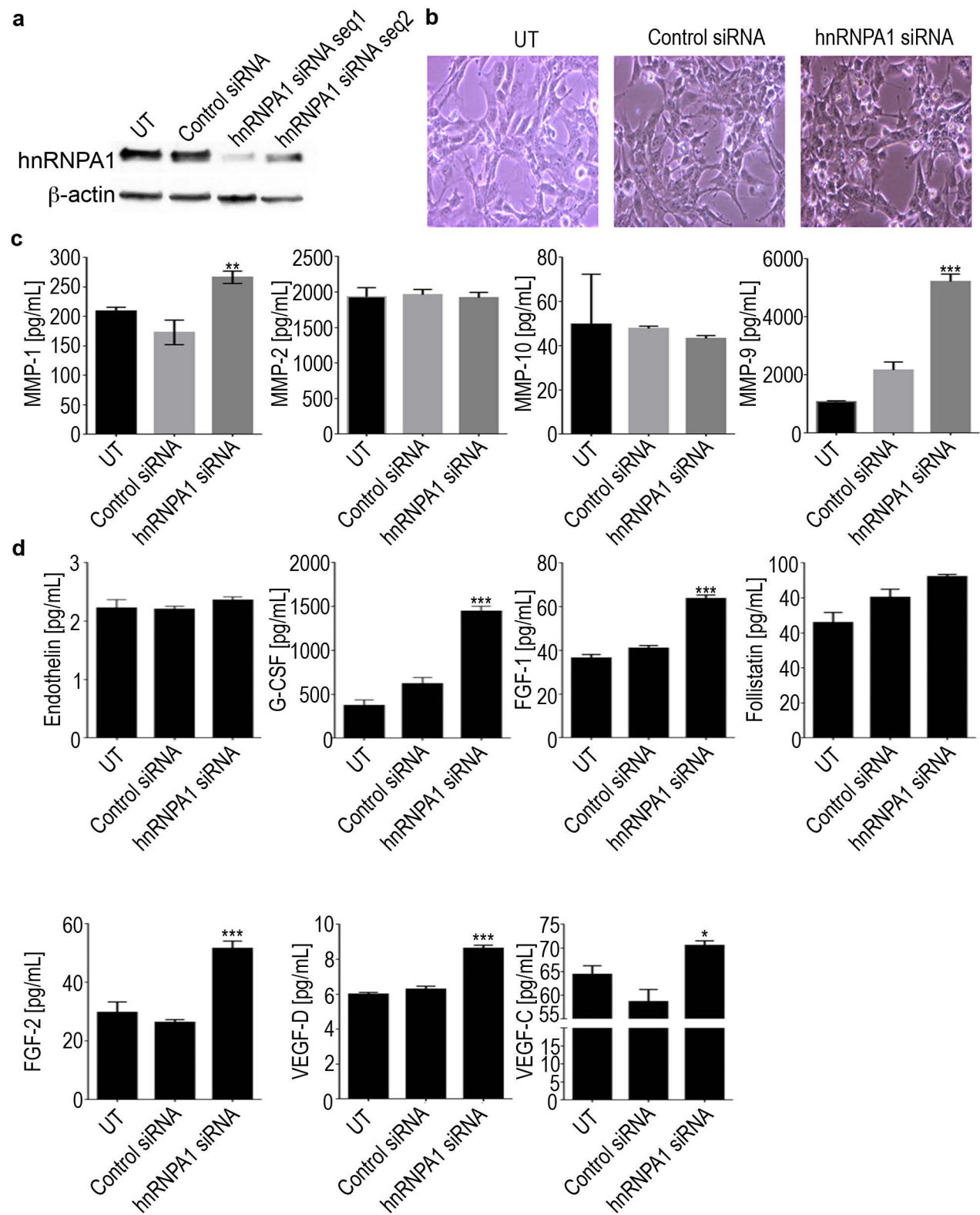

Supplement: Supplementary Figure S3 [file celldisc201729-s3.pdf]

Supplementary Figure S4

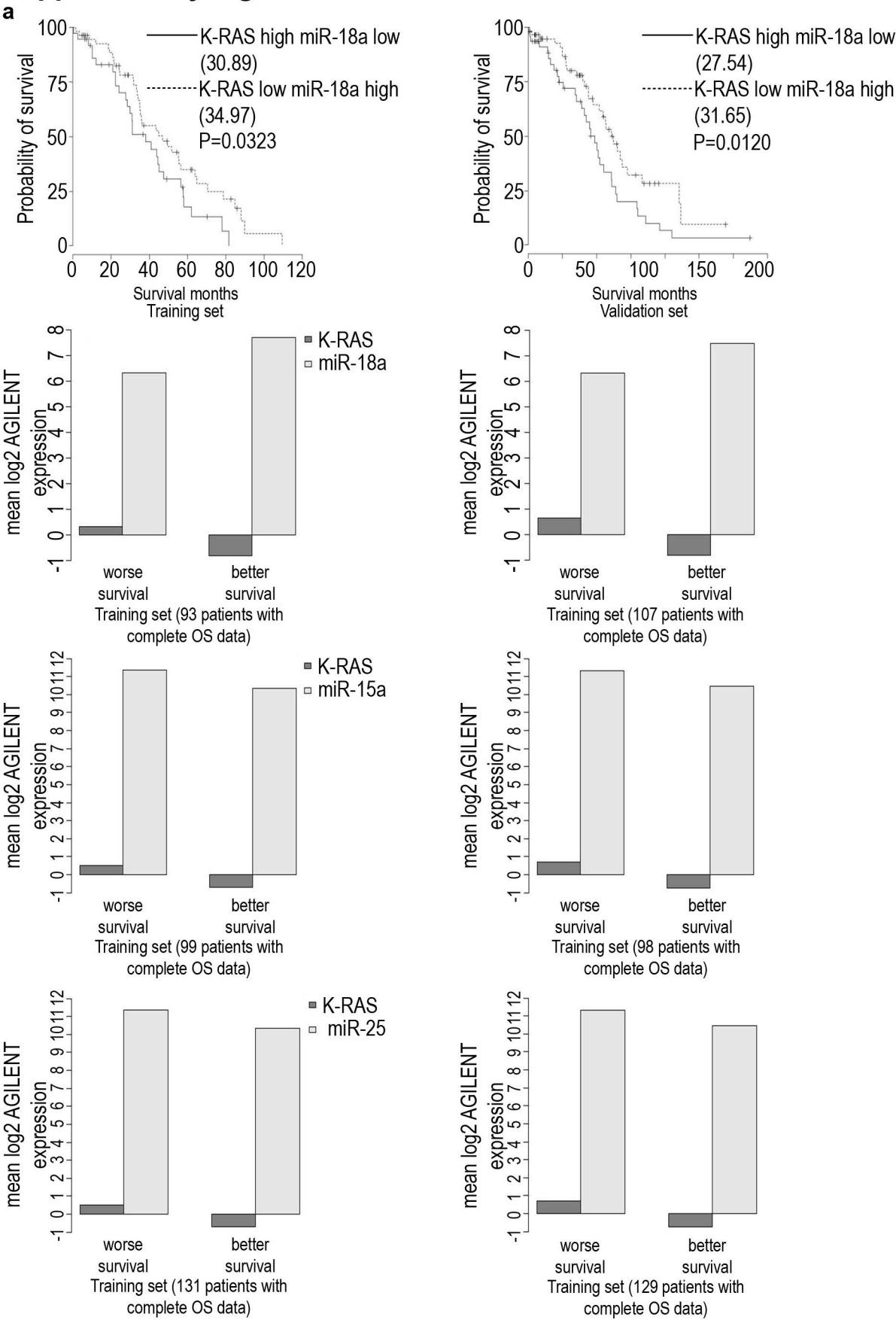

Supplement: Supplementary Figure S4 [file celldisc201729-s4.pdf]
